# Pectin-Based Active and Smart Film Packaging: A Comprehensive Review of Recent Advancements in Antimicrobial, Antioxidant, and Smart Colorimetric Systems for Enhanced Food Preservation

**DOI:** 10.3390/molecules30051144

**Published:** 2025-03-03

**Authors:** Nurul Saadah Said, Won Young Lee

**Affiliations:** 1 School of Food Science and Technology, Kyungpook National University, Daegu 41566, Republic of Korea; nurulsaadah.said@gmail.com; 2 Research Institute of Tailored Food Technology, Kyungpook National University, Daegu 41566, Republic of Korea

**Keywords:** pectin film, active packaging, smart packaging, antioxidant, antimicrobe, color indicator

## Abstract

This review provides a comprehensive overview of recent advancements in biodegradable active and smart packaging utilizing pectin from various origins for food applications. It critically examines the challenges and limitations associated with these developments, initially focusing on the structural influences of pectin on the properties of packaging films. Methods such as spray drying, casting, and extrusion are detailed for manufacturing pectin films, highlighting their impact on film characteristics. In discussing active pectin films, the review emphasizes the effectiveness of incorporating essential oils, plant extracts, and nanoparticles to enhance mechanical strength, moisture barrier properties, and resistance to oxidation and microbial growth. Smart biodegradable packaging is a significant research area, particularly in monitoring food freshness. The integration of natural colorants such as anthocyanins, betacyanins, and curcumin into these systems is discussed for their ability to detect spoilage in meat and seafood products. The review details the specific mechanisms through which these colorants interact with food components and environmental factors to provide visible freshness indicators for consumers. It underscores the potential of these technologies to fulfill sustainability goals by providing eco-friendly substitutes for traditional plastic packaging.

## 1. Introduction

The global food packaging industry stands in critical circumstances, facing the dual challenge of meeting increasing demands for extended food shelf life while urgently addressing the environmental impact of plastic waste. This dilemma has sparked a surge in research toward sustainable, biodegradable packaging materials that aim to match or surpass the performance of traditional plastics. Among these innovative solutions, pectin has emerged as a good candidate, offering a unique blend of functionality, eco-friendliness, and economic viability [1]. Pectin, a complex polysaccharide found in the cell walls of terrestrial plants, is characterized by its primary backbone of α-(1,4)-linked D-galacturonic acid residues [2]. This molecular structure confers pectin with remarkable versatility, making it an ideal candidate for advanced food packaging applications. What sets pectin apart is its inherent properties and its sustainable sourcing: it can be extracted from food industry waste, particularly citrus peels and apple pomace, transforming what would be discarded by-products into valuable packaging materials. The extraction of pectin has significantly evolved, employing methods ranging from conventional acid extraction to cutting-edge techniques like supercritical water, ultrasound, and microwave-assisted extractions [3]. These advancements have not only improved the efficiency of pectin extraction but also enhanced the quality and functionality of the resulting material, opening new avenues for its application in food packaging.

In recent years, environmental concerns and the demand for extended shelf life have driven growing interest in sustainable and functional food packaging solutions. These innovative systems go beyond passive containment, actively interacting with food products to extend shelf life and maintain quality. Active packaging systems incorporating pectin have shown remarkable potential in inhibiting microbial growth, reducing oxidation, and preserving food freshness. Researchers have explored integrating essential oils, plant extracts, and nanoparticles into pectin-based films, enhancing their antimicrobial and antioxidant properties [4,5,6].

This review paper distinguishes itself from previous works by focusing on the cutting-edge application of pectin in smart film packaging, representing a significant leap forward in food preservation technology. While prior reviews have extensively covered pectin’s structure and functional properties in pectin film as active packaging [7,8,9], this paper breaks new ground by exploring its role in smart packaging systems. Key differentiating aspects include a comprehensive analysis of pectin-based smart packaging that enables real-time monitoring of food conditions throughout the supply chain, highlighting the integration of advanced sensing technologies like pH-responsive indicators and freshness sensors directly into pectin-based films, surpassing traditional applications and addressing a gap in earlier literature. Additionally, the review provides an in-depth examination of how natural colorants in pectin-based films react to pH shifts during food deterioration, offering visual indications of freshness. The innovative use of integrated sensors that respond to specific volatile compounds released during meat and seafood spoilage is also explored, providing clear visual cues regarding food quality [10,11,12]. This technology allows consumers to assess food quality and safety without opening the package, significantly enhancing food preservation and reducing waste.

In conclusion, the review emphasizes pectin’s potential to address food packaging and preservation challenges and the environmental and economic implications of adopting pectin-based solutions. As research and innovation continue to refine pectin’s applications and overcome existing limitations, the future holds exciting prospects for integrating this natural polymer into mainstream packaging solutions worldwide.

## 2. Pectin Structure and Physicochemical Properties

**Figure 1:**
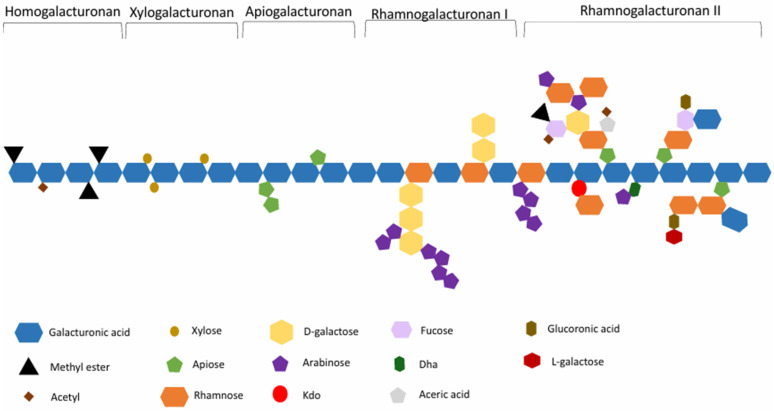
Molecular structure of pectin in plant cell wall.

Pectin, a complex heteropolysaccharide found in plant cell walls, particularly in the middle lamella as depicted in Figure 1, has been the subject of extensive research due to its importance in various industries, including food, pharmaceuticals, and biomedicine. The structure of pectin is characterized by a primary backbone of α-(1,4)-linked D-galacturonic acid residues, forming the homogalacturonan (HG) region. This backbone can be interrupted by rhamnose residues, creating rhamnogalacturonan I (RG-I) regions, while highly branched regions known as rhamnogalacturonan II (RG-II) and xylogalacturonan (XG) contribute to its structural complexity. The HG backbone is partially methyl-esterified at the C-6 carboxyl groups and may also be O-acetylated at the O-2 or O-3 positions, with the degree of methyl-esterification (DM) being a crucial factor in determining pectin’s functional properties [13]. Pectins are classified as high-methoxyl (HM) or low-methoxyl (LM) based on their DM, with HM pectins having a DM > 50% and LM pectins having a DM < 50% [14].

The physicochemical properties of pectin are diverse and largely influenced by its structural characteristics. One of the most important properties is its ability to form gels, with HM pectins forming gels in acidic conditions and high sugar concentrations, while LM pectins can gel in the presence of divalent cations, particularly calcium, over a wider pH range [15]. In solution form, pectin exhibits non-Newtonian, pseudoplastic behavior, with viscosity influenced by factors such as concentration, molecular weight, pH, and temperature [16]. Some pectin types, particularly those with high protein content or specific structural features, can act as emulsifiers, stabilizing oil-in-water emulsions [17]. This rheological behavior is essential in understanding pectin’s performance in various processing conditions and end-use applications. Pectin’s functional versatility is further enhanced by its ability to interact with other molecules. It can form complexes with polysaccharides, proteins, and multivalent cations, leading to various potential applications. In the food industry, pectin finds applications as a gelling agent for jams and jellies, a thickener and stabilizer in various products, an emulsifier, a fat and sugar replacer in low-calorie foods, and a functional ingredient in fruit products, dairy products, desserts, frozen foods, bakery products, beverages, confectionery, and salad dressings [18].

## 3. Pectin as Film Packaging

Beyond these conventional food applications, pectin’s unique properties have garnered significant attention for its use in sustainable food packaging materials. Its biodegradability, edibility, film-forming ability, and versatility, in combination with other materials, make pectin an attractive option for developing eco-friendly alternatives to conventional, non-biodegradable plastics in certain applications. Pectin-based films exhibit excellent oxygen barrier properties, making them particularly suitable for packaging fresh fruits and vegetables. These films can effectively reduce respiration rates and extend the shelf life of perishable produce. For instance, pectin films have been successfully used to delay ripening and maintain the quality of avocados, pears, tomatoes, and other fruits [19,20]. The film’s ability to control gas exchange also helps preserve packaged foods’ color, texture, and nutritional value.

The structure–function relationship in pectin-based films is essential for determining their properties. Different sources of pectin, along with their GA and DE content, are summarized in Table 1, highlighting their respective physical, mechanical, and functional properties. Recent studies reveal that specific structural characteristics of pectin, particularly galacturonic acid (GA) content and degree of esterification (DE), significantly influence film performance. Higher GA content enhances film strength and flexibility while also affecting moisture barrier properties [21]. A study by Said et al. [22] found a strong positive correlation (r = 1.0) between DE and mechanical properties in films from hybrid citrus peels, indicating that esterification patterns significantly affect film strength and flexibility. DE also influences the oxygen barrier performance of pectin films, reinforcing its importance in the design of packaging materials. Moreover, the monosaccharide composition of pectin, which affects both mechanical and barrier properties, shows strong correlations (r = 0.8–1.0) with these characteristics.

Based on the data from Table 1, low DE pectin films (DE < 50%) exhibit higher elongation at break (EAB), making them suitable for applications requiring flexibility and stretchability with lower TS reported. For instance, plantain peel pectin films have the highest EAB at 442%, despite having a very low TS of 0.04 MPa, indicating a highly flexible but weak film [23]. Similarly, low-grade sun-dried fig pectin films present moderate TS (3.11 MPa) and EAB (15.19%), making them ideal for coatings requiring both flexibility and moderate strength [21]. Stalk waste-derived fig pectin films, with a DE of 45%, exhibit an improved TS of 6.50 MPa and higher EAB (26.17%), suggesting that a slight increase in DE enhances film toughness while maintaining flexibility [21]. *Tiliacora triandra* pectin films (DE 48.36%) have a TS of 7.10 MPa and an EAB of 7.17%, while the addition of nanochitosan results in a TS of 2.86–10.84 MPa and an EAB of 8.65–17.22%, showing an improvement in mechanical flexibility [24]. In addition to that, a comparative study between low methyl-esterified (LM) and high methyl-esterified (HM) citrus pectin films showed that LM citrus pectin films (L200 and L201Y) exhibit lower TS values (21.15–23.85 MPa) with EAB (2.59–3.88%). Conversely, in the same study, high methyl-esterified citrus pectin (H182) demonstrated an even greater TS (30.61 MPa) while maintaining a similarly low EAB (3.93%) [25]. Higher Mw and DM in HM pectin result in longer polymer chains, increasing intermolecular entanglement and solution viscosity, enhancing the resulting film’s strength and flexibility [26]. This structural entanglement contributes to improved TS and EAB, forming a dense, cohesive film network. However, despite their adaptability for food wrapping, low DE pectin films tend to be mechanically weaker and more sensitive to humidity, leading to faster degradation in wet conditions.

In addition, incorporating or blending pectin with other biopolymers or substances significantly impacts film properties, providing additional versatility for tailoring pectin-based films for diverse applications. Blending pectin with natural biopolymers such as carboxymethyl cellulose (CMC), chitosan, or starch often yields synergistic effects that enhance film properties [27]. For instance, pectin/CMC blends have demonstrated improved mechanical properties compared to pure pectin films [28]. Furthermore, as observed in chitosan–pectin bilayer films, polyelectrolyte assembly significantly enhances mechanical properties [25]. The electrostatic interactions between the positively charged chitosan and negatively charged pectin, combined with hydrogen bonding between functional groups, strengthen the film structure [26]. Adding proteins like whey or soy protein isolate can enhance mechanical strength and water resistance, creating more compact film structures with better barrier properties [29]. Plasticizers such as glycerol or sorbitol are commonly used to improve flexibility and reduce brittleness, with their type and concentration significantly affecting mechanical properties and water vapor permeability [30]. Lipid incorporation, using substances like beeswax or fatty acids, greatly improves water vapor barrier properties, though it may reduce transparency [31]. Cross-linking agents like Ca^2+^, Cu^2+^, Fe^2+^, or Zn^2+^ ions or citric acid can improve mechanical and barrier properties, resulting in stronger, less water-soluble films [32]. These enhancements offer a broad spectrum of functionality for pectin-based films, optimizing them for various packaging and preservation need.

Compared to protein films, pectin films generally have good tensile with less flexibility. Low-DE pectin films, such as pineapple peel pectin (TS 3.07–5.60 MPa, EAB 5.66–14.84%) [33], demonstrate lower mechanical integrity, while high-DE citrus pectin (H182) reaches a TS of 30.61 MPa with a low EAB of 3.93% [25]. This was similar with zein films, which exhibit TS values of 21.45 MPa but with better stretchability (EAB 34.88%) [34]. SPI films range from 2.60–10.83 MPa in TS [35], while wheat gluten films offer higher mechanical integrity (TS 4.1–11.7 MPa, EAB 44.50–175.4%) [36,37], making them less stronger than most pectin films. In terms of barrier properties, films exhibit strong oxygen and lipid barrier properties, reducing oxidation and preserving food quality [38]. Their oil-, grease-, and odor-proofing capabilities further enhance shelf life by preventing volatile compound migration. Pectin-based films outperform cellulose and starch due to their superior gelling and film-forming properties. Their high water-retention capacity and transparency make them ideal for moisture-sensitive coatings, effectively maintaining food freshness. The addition of polysaccharide or protein polymers to pectin film composition as bilayer or composite film can significantly improve mechanical strength and elasticity, making it a viable alternative for food packaging.

**Table 1:** Types of pectin sources, structure, and film properties.

Pectin Film Type	Pectin Yield (%)	GA	DE (%)	Film Properties					References
				Thickness	WVP	Mechanical	Antioxidant	Antimicrobial	
Pineapple peel pectin	1.02–2.12	35.30–44.78%	29.92–39.39	-	2.16–3.83 × 10^−10^ gm^−1^ s^−1^ Pa^−1^	TS-3.07–5.60 MPa EAB-5.66–14.84% YM-75.21–152.09 MPa	DPPH-15.53–137.00 µM Trolox/g film ABTS-47.95–936.45 µM Trolox/g film TPC-33.64–113.65 mg GAE/g film	-	[33]
Plantain peel pectin	12.04–13.38	62.0–123.2 mg	43.3–78.8	-	41.31 × 10^−7^ gm^−1^hPa	TS-0.04 MPa EB-442% YM-6.78 MPa	-	-	[23]
Low-grade sun-dried figs pectin	-	32.2%	34.2	89.02 µm	30.39 g.mm.m^−2^·day^−1^·kPa^− 1^	TS-3.11 MPa EB-15.19% YM-0.55 MPa	-	-	[21]
Stalk wastes of sun-dried figs pectin	-	36.7%	45.0	84.11 µm	31.74 g.mm.m^−2^·day^−1^·kPa^−1^	TS-6.50 MPa EB-26.17% YM-0.70 MPa	-	-	[21]
Grapefruit peel pectin	48.35	-	78.38	95.0 µm	1.13 × 10^−10^ g.m^−1^·s^−1^·Pa^−1^	TS-15.09 MPa EB-19.12%	-	Reduced 0.7 log units of *E. coli* O157:H7 compared to non-coated food sample	[39]
Mango peel pectin	21.82	-	-	-	1.34–1.61 × 10^−10^ g/s m^−1^ Pa^−1^	TS-11.04–14.57 MPa EB-24.51–33.71% YM-117.98–311.94 MPa	DPPH-15.01–68.36 TPC-244.34–511.31 mg GAE 100 g^−1^ film	-	[40]
Watermelon rind pectin	-	-	-	0.081 mm	4.00 × 10^−9^ g/m·s·Pa)	TS-42.30 MPa EB-10.77% YM-393.91%	DPPH-2.90% ABTS-6.77% FRAP-0.10 mM Fe (II)/g film TPC-2.14 mg GAE/g film	-	[41]
Pumpkin peel pectin	69.89	-	-	0.18 mm	5.09 × 10^−6^ g/Pa·m·h	TS-5.28 MPa EB-14.37%	-	-	[42]
Watermelon peel pectin	-	-	-	89.15 µm	3.02–5.72 × 10^−8^ g·mm·kPa^−1^s^−1^m^−2^	TS-6.33 MPa EB-35.44%	-	-	[43]
White pomelo peel pectin	-	-	-	0.107 mm	0.95 × 10^−12^ g/Pa·h·m or 2.63 × 10^−16^ g/m·s·pa	TS-15.58 MPa EB-5.91%	-	-	[44]
Fresh white-fleshed pitaya (*Hylocereus undatus*) peel pectin	20.86	1.22	46.06	42.32 µm	2.00 × 10^11^ g·m^−1^·s^−1^·Pa^−1^	EAB-18.32%	-	-	[45]
Dragon fruit peel pectin	18.00	-	36.00	-	-	TS-2.50–5.00 MPa EAB-8.33–13.33%	-	-	[46]
Banana peel pectin	-	-	-	0.0311–0.0387 mm	-	TS-2.63–10.56 MPa EAB-16.66–58.33%	-	-	[47]
*Citrus junos* pomace pectin	-	-	-	0.078 mm	4.20 × 10^−9^ g/m·s·Pa	TS-22.91 MPa EAB-10.42% YM-223.93 MPa	TPC-5.08 mg GAE/g film Reducing power-1.48 mg ascorbic acid/g film FIC-35.14%	-	[48]
Citrus pectin	-	-	-	0.06 mm	13.39 × 10^−11^ g/m·s·Pa	TS-14.08 MPa EAB-7.14%	-	-	[49]
Citrus pectin	-	-	-	0.057 mm	13.22 × 10^−11^ g·m^−1^·s^−1^·Pa	TS-14.78 MPa EAB-6.37% YM-2.32	TPC-0.49 mg gallic acid/g	-	[5]
Low methyl-esterified citrus pectin (L200)	-	-	26.90	0.062 mm	0.99 × 10^10^ g·m^−1^·s^−1^·Pa	TS-21.15 MPa EAB-2.59%	-	-	[25]
Low methyl-esterified citrus pectin (L201Y)	-	-	26.50	0.063 mm	0.99 × 10^10^ g·m^−1^·s^−1^·Pa	TS-23.85 MPa EAB-3.88%	-	-	[25]
High methyl-esterified citrus pectin (H183)	-	-	68.90	0.065 mm	1.06 × 10^10^ g·m^−1^·s^−1^·Pa	TS-24.64 MPa EAB-3.96%	-	-	[25]
High methyl-esterified citrus pectin (H182)	-	-	69.50	0.070 mm	1.10 × 10^10^ g·m^−1^·s^−1^·Pa	TS-30.61 MPa EAB-3.93%	-	-	[25]
Citrus pectin/clove bud essential oil	-	-	-	0.068–0.094 mm	6.52–9.48 ×10^−11^ g·m^−1^·s^−1^·Pa	TS-209.98–33.78 MPa EAB-8.96–11.75% YM-2.34–2.87	DPPH-5.80–64.73% TPC-13.52 mg gallic acid/g	Inhibitory effects against *E. coli*, *L. monocytogenes*, and *S. aureus*	[5]
Orange peel pectin/dittany; Orange peel pectin/anise	9.67–19.30	-	70.0–75.0	Dittany; 39.0–41.0 µm Anise; 39.0–40.0 µm	-	Max force-Dittany; 2.04–3.08 N YM-91.30–191.80 kPa Anise; Max force-2.00–4.65 N YM-76.62–155.09 kPa	DPPH-~62 to 91% TPC-0.035 to 0.157 mg GAE/0.5 mL	-	[50]
Hawthorn fruit pulp pectin/candelilla wax	4.30	-	76.90	63.00–94.30	1.30–9.30 × 10^14^ mol·m·s^−1^·m^−2^·Pa^−1^	TS-0.16–0.22 MPa EB-57.20–4.40%	-	-	[51]
Blood orange peel pectin + grey triggerfish skin gelatin	30.78	81.2%	-	97.36–98.64 µm	1.54–1.85 × 10^−10^·g·m^−1^·s^−1^·Pa^−1^	TS-7.80–14.36 MPa EB-4.36–7.02%	DPPH-35.90–67.36% Reducing power (OD_700_)-0.38–0.96 β-carotene bleaching inhibition-37.92–50.36%	-	[52]
*Cissampelos pareira* leaves pectin/PVA	33.00–35.00	-	-	42.00–71.00 µm		TSW-4.00–9.40 MPa EAB-14.00–22.00% YM-14.90–53.40 MPa	DPPH-28.00–84.00%	-	[53]
Mango peel pectin/SiO_2_	3.50–31.70	-	15.50–50.20	0.28 mm	3.05 g.mm^−1^m^2^·KPa·day	TS-36.77–61.31 MPa EB-6.29–9.32% YM-6.81–16.49 MPa	-	-	[54]
Jerusalem artichoke residue pectin/chitosan	10.00	-	-	<42.00 µm	>3.37 × 10^−11^ g·m^−1^·s^−1^·Pa^−1^	-	-	-	[55]
Ripe plantain peel pectin/PVA	40.05	2.89 mg/mL	59.52	-	-	TS-1.43–5.51 MPa EAB-3.13–5.75%	-	-	[56]
*Tiliacora triandra* pectin	-	-	48.36	54.00	1.33 g·mm/m^2^·day·kPa	TS-7.10 MPa EAB-7.17%	-	-	[24]
Tiliacora triandra pectin/nanochitosan	-	-	48.36	43.00–61.20	0.1755–0.5922 g.mm/m^2^·day·kPa	TS-2.86–10.84 MPa EAB-8.65–17.22%	-	Antibacterial activity against *E. coli*, *S. cerevisiae*, *A. niger*, and *C. gloeosporioides*	[24]
Fresh white-fleshed pitaya (*Hylocereus undatus*) peel pectin/montmorillonite	20.86	1.22	46.06	43.15–43.78 µm	1.65–1.94 × 10^11^ g·m^−1^·s^−1^·Pa^−1^	EAB-14.28–17.76%	-	-	[45]
Yellow passion fruit pectin/fish myofibrillar protein	-	5.37%	88.95	0.022 mm	2.08–3.52 × 10^−13^ g/m·s·Pa	TS-1.83–12.20 MPa EAB-264.87%	-	-	[57]
Passion fruit peel pectin/ chitosan	16.16	62.92%	50.74	0.100 mm	1.20 g·mm/h·m^2^·kPa	TS-17.67 MPa EAB-23.27% YM-450.09%	-	Antibacterial activity against *P. aeruginosa*, *S. aureus*, *K. pneumoniae,* and *B. cereus*	[58]
Passion fruit peel pectin/ chitosan/ *Piper betle* L. leaf extract	16.15	62.92%	50.74	0.177–0.213 mm	1.34–1.77 g·mm/h·m^2^·kPa	TS-10.09–16.91% EAB-16.06–28.03% YM-200.05–366.22 MPa	-	Antibacterial activity against *P. aeruginosa*, *S. aureus*, *K. pneumoniae* and *B. cereus*	[58]
Mango peel pectin/low methoxyl pectin	10.33	67.95%	76.47–79.00	30.00–36.00 µm	-	TS-4.98 MPa EAB-8.80% YM-83.19 MPa	-	-	[59]

### 3.1. Fabrication Method of Film Packaging

**Figure 2:**
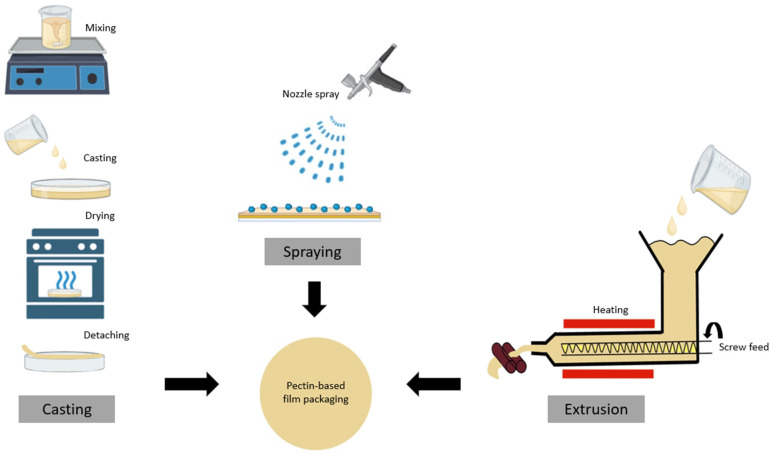
Schematic diagrams for film formation methods of pectin-based films.

Film formation methods are crucial in determining the packaging material’s final properties and commercial viability when developing pectin-based active packaging. These methods can be broadly categorized into wet and dry processes, each with its own set of techniques and characteristics. The wet process encompasses casting and spraying methods, while the dry process primarily refers to extrusion techniques, as demonstrated in Table 2 and schematically visualized in Figure 2.

#### 3.1.1. Spraying Method

The film spraying method is a wet process technique employed in developing films for food packaging applications, operating on the principle of atomizing and spraying a film solution onto a surface to form a thin, uniform film [60,61]. This process involves several key steps, beginning with preparing a film solution by dissolving biopolymers in a suitable solvent, typically water or a water-based mixture, along with any necessary additives such as plasticizers or functional compounds. The prepared solution is then atomized and sprayed onto a substrate or surface using specialized equipment, where the sprayed droplets coalesce to form a continuous liquid film. Subsequently, the sprayed film undergoes a drying process, usually through evaporation, to solidify into a packaging film [62].

Developing pectin films through spraying requires careful consideration of various factors, including solution formulation, spraying parameters, substrate selection, and drying conditions. The pectin concentration and type are optimized for spraying, while plasticizers like glycerol may be added to enhance film flexibility [63]. Spraying parameters such as nozzle type and size, spray pressure, distance from the substrate, spray pattern, and speed are adjusted to achieve desired film properties and uniformity. The choice of substrate can significantly affect film adhesion and final properties, with non-stick surfaces often used for easy film removal. Controlled drying conditions are crucial for obtaining uniform films, with temperature, humidity, and airflow during drying influencing the final film characteristics [64]. The spraying method offers several advantages in pectin film development, including the ability to produce very thin, uniform films, effectively coat irregular surfaces, and easily control film thickness. However, it also presents challenges such as the need for specialized equipment, potential material waste due to overspray, and difficulty achieving consistent thickness and properties across large areas [65].

Recent developments in food packaging research suggest potential areas of advancement in pectin film spraying, including the inclusion of nanoparticles or bioactive compounds and the creation of multilayer films through sequential spraying of different biopolymer solutions [66] to enhance the film properties. Despite its challenges, the film spraying method remains valuable in the ongoing research and development of advanced pectin-based films, contributing to the evolution of sustainable food packaging solutions.

#### 3.1.2. Casting Methods

The film casting process, also known as the solvent-casting method, is a widely used wet process technique for developing pectin-based films in food packaging applications. This method is based on the principle of dissolving pectin in a suitable solvent, typically water or water-based, followed by incorporating necessary additives such as plasticizers, antimicrobial agents, or other polymers. The resulting solution is then cast onto a flat surface, often a glass plate or non-stick surface, and allowed to dry under controlled conditions to form a solid film [67]. This process allows for developing pectin films with various properties and functionalities, depending on the composition of the casting solution and the drying conditions employed. The casting method offers several advantages, including its simplicity, flexibility in incorporating different additives, and suitability for laboratory-scale research and development of new film formulations. However, it also presents challenges, particularly regarding scalability for large-scale production, ensuring complete solvent removal, and achieving consistent film thickness and properties [68].

Recent developments in pectin film casting have focused on creating composite or layering films, which have improved mechanical properties compared to pure pectin films [69]. Additionally, researchers have been working on optimizing casting conditions to produce pectin-based films with reduced thickness, lower water vapor transmission rates, and higher transparency [70]. The casting method has also been instrumental in developing active packaging solutions by incorporating bioactive compounds into pectin films. Despite its limitations in scalability, the film casting process remains an essential technique for researching and developing new pectin-based film formulations with enhanced properties and functionalities, significantly contributing to the advancement of sustainable food packaging solutions.

#### 3.1.3. Extrusion Method

The dry process, exemplified by extrusion, represents a continuous method where pectin, often combined with other polymers or additives, is introduced into an extruder, heated, mixed thoroughly, and then extruded through a die to form films or sheets [71]. Extrusion offers superior efficiency over wet processes and is particularly well-suited for large-scale commercial production, providing precise control over film properties and ensuring consistent characteristics necessary for industrial manufacturing. The choice between extrusion and wet methods depends on desired film properties, production scale, available equipment, and specific application requirements. While casting and spraying methods are commonly utilized in research and development due to their flexibility in incorporating active compounds, extrusion is favored for commercial-scale production of pectin-based active packaging films due to its efficiency and scalability [72]. Extrusion’s advantages also include continuous processing, environmental sustainability by eliminating solvent use, and versatility in accommodating various additives, resulting in films with superior consistency compared to wet methods.

Inside the extruder, pectin undergoes heating and intensive mixing. It is forced through a die under controlled parameters such as temperature (typically 80–140 °C), screw speed, and pressure, which are crucial for achieving uniform film properties. Die design, typically employing flat dies, enables precise film thickness and width adjustment. Post-extrusion treatments such as stretching and surface modifications further enhance the film’s mechanical strength and barrier properties [73]. However, challenges exist in the extrusion process of pectin-based films, notably in managing pectin’s thermal sensitivity to prevent molecular degradation and optimizing rheological properties for efficient extrusion. To address these challenges, researchers and manufacturers have developed several strategies. Reactive extrusion involves chemical modifications of pectin during the extrusion process, allowing for tailored film properties through carefully controlled reaction conditions [74]. Co-extrusion with other biopolymers like starch or polyvinyl alcohol (PVA) can lead to multifunctional films with improved mechanical strength, barrier properties, or processability compared to pure pectin films [75]. Additionally, precise temperature management throughout the extrusion process is critical to prevent pectin degradation while ensuring proper melting and flow. The structure of pectin, including its degree of methylation, relates to its behavior during extrusion and significantly influences the process parameters required for successful film formation. Moreover, incorporating plasticizers can improve pectin’s processability and lower the processing temperature, reducing the risk of thermal degradation [76]. In addition, specialized screw designs can help manage shear and mixing to achieve uniform pectin melts without excessive degradation [77].

**Table 2:** Film formation methods for pectin-based films.

Film Preparation Methods		Type of Film	Preparation Condition	Reference
Wet process	Spraying	Pectin/chitosan	Two parallel nozzles onto a rotating cylinder Air Pressure: 3 bar Flow Rate: 3.0 mL/min Distance: 20 cm Drying: 60 °C during and 30 min post-spraying	[78]
	Spraying	Citrus peel pectin	Electrospraying process uses an electric field Flow Rate: 4 mL/h Distance: 4.5–6.0 cm Drying: Hot air temperature (150 °C)	[62]
	Spraying	Pectin/zein	Rotary drum collector Flow Rate: 4.0–14.5 mL/h Distance: 1.5–4.5 cm (injection point to drum) Drying: Hot air at 150 °C, preheating drum to 80 °C	[66]
	Casting	Dragon fruit peel pectin	Pectin in distilled water (2%, *w*/*v*) Plasticizer Addition: Glycerol (30%, *w*/*w*) Method: Poured into Petri dish and dried 45 °C for 24 h	[79]
	Casting	Hybrid citrus peel pectin/PLA	Pectin in distilled water (1.96%, *w*/*v*) Plasticizer Addition: Glycerol (30%, *w*/*w*) Method: Poured into Petri dish, bilayer with PLA solution and dried 45 °C for 48 h	[80]
	Casting	Coffee waste pectin/chitosan	Pectin solution added to chitosan solution Method: Poured into Petri dish and dried for 72 h at room temperature	[81]
Dry process	Extrusion	HM pectin	Extruder: Co-rotating twin-screw extruder (model SJSL 20) Screw Diameter: 20 mm, L/D ratio: 40 Temperature profile: 35, 50, 75, 95, 100, 100, 90 °C (from feeder to matrix) Screw Speed: 100 rpm Pelletizer Speed: 120 rpm Pellet Size: 2 mm	[82]
	Extrusion	Pectin/starch	Equipment: Werner Pfleiderer ZSK30 twin screw extruder Flow Rates: 27–102 g/min (1.62–6.12 kg/h) Feed Rates: 0.43–2.85 mL/g Moisture: 30–75% Temperature Profiles: Six different profiles Screw Speeds: 350 rpm (Trials 1–3), 350, and 450 rpm (Trial 4)	[76]
	Extrusion (Two-stage)	Pectin/starch/ PVA	Equipment: Werner-Pfleiderer ZSK30 twin screw extruder (Krupp, Werner-Pfleiderer Co., Ramsey, NJ, USA). Screw Speeds: 20 rpm (film blowing), 30 rpm (sheet extrusion) Torque: 20–90 Nm Temperature Profile: 35, 35, 50, 75, 110, 120, 100, 100, 90 °C (zones 1 to 9)—condition 1 35, 35, 50, 75, 95, 100, 100, 85, 70 °C (zones 1 to 9)—condition 2	[75]

### 3.2. Active Film Packaging

Active film packaging represents an innovative approach to food preservation, incorporating functional additives or compounds into packaging materials to create a dynamic interaction between the package, the product, and the environment [83]. This method, which aims to extend shelf life, improve food safety, and enhance food quality, operates on the principle of integrating active agents into a polymer matrix, such as pectin, to facilitate interaction with the food or its surroundings. The mechanism of active film packaging typically involves one or more processes, including the gradual release of active compounds like antimicrobials, antioxidants, or flavors into the food or package headspace; the absorption or scavenging of undesirable compounds such as oxygen, ethylene, or moisture from the package environment; and the controlled permeability of gases or moisture to maintain optimal conditions inside the package. The development of active films begins with selecting a suitable polymer as the film matrix, followed by incorporating active agents into the polymer solution or melting during film formation. These active agents can include essential oils, plant extracts, metal nanoparticles, and other substances aiding in preserving the food quality, as shown in Table 3. The mixture is then formed into a film using methods such as casting, extrusion, or spraying, after which the films are analyzed for their physical, chemical, and functional properties.

#### 3.2.1. Essential Oil

Essential oils are promising active ingredients in food packaging films, offering natural antimicrobial and antioxidant properties that protect food products. Extracted from plants, these volatile, aromatic compounds consist of terpenes, terpenoids, and other aromatic and aliphatic compounds, which preserve food quality through various mechanisms [102]. The primary mechanisms include antimicrobial activity, where essential oils inhibit or slow down the growth of microorganisms by disrupting cell membranes, interfering with cellular energy production, altering cell structure, and inhibiting enzyme systems [103]. Additionally, essential oils have antioxidant effects, with many containing compounds that can scavenge free radicals and prevent oxidation reactions in foods [104]. The film matrix acts as a carrier, allowing for the gradual release of essential oil compounds into the food or package headspace over time. This controlled release, combined with enhanced barrier properties against oxygen and moisture, helps slow degradation processes. The essential oil compounds also inhibit microbial growth on food surfaces and possess antioxidant properties that prevent oxidation. The films also regulate moisture transfer, maintaining optimal food hydration, while helping to retain fresh aromas by controlling volatile compound release.

The incorporation of EOs into pectin-based films modifies mechanical properties by reducing tensile strength (TS) and increasing elongation at break (EAB) due to their plasticizing effect. Low molecular weight EO components disrupt intra- and intermolecular hydrogen bonds, replacing strong polysaccharide interactions with weaker EO–polysaccharide interactions, resulting in a more flexible but less rigid film [105,106]. Additionally, EO molecules increase macromolecular mobility, facilitating polymer chain movement and thereby enhancing film stretchability. The plasticizing effect is further influenced by the presence of emulsifiers such as glycerol or Tween 80, which weaken intermolecular interactions and promote phase separation within the film, further reducing TS [107,108,109]. The impact of EOs on mechanical behavior is highly dependent on their type, concentration, and the composition of the film formulation. For applications demanding higher mechanical resistance, careful formulation adjustments, such as reducing EO concentration or introducing reinforcing agents like nanoclays or bilayering with other bioploymer, are necessary to counteract the loss of TS while maintaining the functional benefits of EO incorporation.

The incorporation of EOs into pectin-based films improves their stability when in contact with high-moisture foods by enhancing hydrophobicity, reinforcing the polymer structure, and regulating moisture exchange, which can be attributed to the presence of bioactive compounds such as polyphenols, flavonoids, and tannins. These compounds possess hydrophobic properties, which increase the water contact angle by modifying the surface chemistry of the film, thereby reducing direct interaction with water molecules [110]. While polyphenols and tannins can form hydrogen bonds with cellulose, decreasing hydrophilicity, hydrophobic terpenes and flavonoids create a moisture barrier, limiting water diffusion and preventing swelling [111]. Phenolic-rich EOs (e.g., oregano, thyme, cinnamon) interact with pectin molecules, forming a denser polymer matrix that slows water penetration, whereas plasticizing EOs (e.g., orange, lemon) increase flexibility but may reduce mechanical strength. Structural modifications also regulate water vapor permeability (WVP), controlling moisture uptake and preventing film degradation.

Research has demonstrated the efficacy of various essential oils in food preservation when incorporated into packaging films. For instance, clove bud, thyme, and oregano essential oils have shown significant antimicrobial activity against common bacteria (*E. coli*, *P. aeruginosa*, *S. aureus*, and *L. monocytogenes*) when incorporated into pectin-based films [5,88]. Incorporating essential oils into pectin films creates an antimicrobial packaging material that inhibits common foodborne pathogens, enhancing food safety and extending shelf life. For example, summer savory essential oils in pectin films have been shown to extend the shelf life of chicken fillets by reducing pH levels and controlling volatile compounds, creating an environment less favorable for microbial growth [91]. This approach not only inhibits bacterial proliferation but also helps maintain the sensory qualities of the food. However, using essential oils in active packaging requires careful optimization to ensure efficacy without compromising food taste or aroma, compatibility with the film matrix, regulatory compliance, and stability of the essential oil compounds over time and under various conditions. Despite these challenges, essential oil–infused pectin films offer a promising solution for natural food preservation in packaging applications.

Essential oils are highly concentrated and should be used with caution. Thyme and oregano oils require dilution and professional guidance, with oregano’s intake limited to 0.27 µg/kg/day due to carvacrol content [112]. Animal studies found no fatalities at 2000 mg/kg bw, though doses above 200 mg/kg bw/day led to feed refusal. The no observed adverse effect level (NOAEL) for oregano oil was established at 200 mg/kg bw/day, confirming its safety at this level [113]. Clove essential oil is generally recognized as safe (GRAS) when consumed at concentrations below 1500 mg/kg [114]. *Thymus capitatus* and *Cinnamomum verum* oils have limited dosage data, though thyme species show a NOAEL above 250 mg/kg/day in animal studies [115]. Citrus essential oil including grapefruit is generally recognized as safe (GRAS) and has been found to be non-irritating and non-sensitizing in human studies. When tested at concentrations of 10% and 100% on volunteers, no irritation or sensitization reactions were reported [116,117]. Summer savory oil has no defined safe dose and should be used cautiously.

#### 3.2.2. Plant Extracts

Plant extracts are one of the active ingredients used in food packaging films, particularly in developing active packaging systems, harnessing their natural bioactive compounds such as polyphenols, flavonoids, and tannins to create a protective environment for food products [118]. These compounds, often possessing antimicrobial, antioxidant, and other beneficial properties, contribute to food preservation through various mechanisms. They inhibit or slow down microbial growth by disrupting cell membranes, inhibiting enzyme systems, interfering with quorum sensing, and chelating essential metals [119]. Additionally, they provide antioxidant effects by scavenging free radicals and preventing food oxidation reactions [118]. Plant extracts also enable the controlled release of bioactive compounds from the pectin film matrix into the food or package headspace over time. Furthermore, they enhance barrier properties against oxygen and moisture, extending packaged products’ shelf life [84].

Beyond their preservative effects, plant extracts also enhance the structural stability of pectin films, reducing their hygroscopic nature. The functional groups in these compounds form intermolecular bonds with pectin, minimizing free hydroxyl (-OH) groups available for water absorption and improving moisture resistance [84]. Their role as natural cross-linking agents strengthens the polymer network, restricting chain mobility and limiting water diffusion, while phenolic and flavonoid compounds introduce a degree of hydrophobicity, further preventing excessive moisture uptake. As a result, the reinforced pectin film acts as a semi-permeable barrier, regulating transpiration, minimizing water loss, and maintaining fruit firmness, effectively preventing shriveling during storage. By modulating gas exchange, these films delay enzymatic and oxidative degradation, helping to preserve the texture and freshness of perishable fruits for a longer duration compared to uncoated produce.

For instance, pectin-based films with neem leaf and mulberry extracts delay spoilage and increase the shelf life of fruits like apricots and bell peppers, while chinar and green tea extracts improve tensile strength and antioxidant activity, and extend the shelf life of fatty food packaging by over 77 days [84,85,86]. Pectin films incorporated with plant extract from neem leaf containing active constituents, including azadirachtin, Nimbin, nimbanene, ascorbic acid, nimbio, and others, demonstrate antimicrobial effects against the bacterial strain of *S. aureus* and the fungal strain of *A. niger* [85]. Green tea extract, rich in catechins, significantly extends the shelf life of fatty foods by preventing rancidity [120]. Barrier properties are enhanced by polyphenols forming cross-links with the pectin film matrix, hydrophobic compounds increasing water vapor barrier properties, and improved mechanical strength through filling interstitial spaces [86]. Incorporating natural extracts into pectin films as active compounds offers benefits but also entails considerations such as volatility, sensory impact, variability in composition, allergenic risks, potential discoloration, mechanical changes, reduced transparency, regulatory challenges, cost implications, heat sensitivity, and compatibility issues with film components. Hence, these drawbacks should be carefully considered during the film development planning process.

For the safety precautions of natural extracts listed in Table 3, mulberry leaf extract (MLE) is considered safe at 250–550 mg daily [121,122], with a NOAEL of 7.50 g/kg body weight/day [123]. Neem extracts exhibit varying toxicity levels depending on formulation and species. Acute toxicity studies report LD₅₀ values ranging from 31.62 mg/kg (leaf extract) to 31.95 g/kg (oil), with dose-dependent effects in animals [124]. Green tea extract (GTE) is safe at >300 mg EGCG daily, though doses exceeding 800 mg/day may increase liver injury risk [125,126]. While green propolis and apple polyphenols lack specific safety data, moderate consumption is generally considered safe. Proper dosing is crucial to ensure food safety while maximizing preservation benefits.

#### 3.2.3. Metal Nanoparticles

As extensively documented in the literature, metal nanoparticles are widely recognized for their functional properties when incorporated into packaging materials. They significantly contribute to food preservation by exhibiting antimicrobial activity and efficiently scavenging oxygen. Nanoparticles such as silver, zinc oxide, and copper are commonly employed, each offering specific mechanisms to enhance food quality [4,98]. Metal nanoparticles, especially silver, display potent antimicrobial properties by disrupting bacterial cell membranes, interfering with DNA replication, and generating reactive oxygen species that damage cellular components. They also scavenge oxygen within the package, slowing food oxidation processes [4]. The pectin film matrix acts as a carrier, facilitating the controlled release of metal ions from nanoparticles into the food or package headspace over time. Additionally, metal nanoparticles enhance the barrier properties of pectin films against oxygen and moisture [93,96]. These mechanisms contribute to food preservation by inhibiting microbial growth on the surface, preventing oxidation processes, and maintaining overall food quality for extended periods.

Incorporating metal nanoparticles into pectin films significantly enhances their performance through various mechanisms, affecting both film properties and functional capabilities. Metal nanoparticles, such as silver and zinc oxide, improve tensile strength by reinforcing the pectin matrix and filling interstitial spaces [95,96]. Their large surface area and binding sites further enhance polymer interaction and controlled NP release, preventing swelling and structural degradation in high-humidity environments. ZnO-based films, particularly those reinforced with crystalline nanocellulose (CNC) or crosslinked with Ca^2+^ ions, exhibited superior tensile strength and enhanced barrier properties due to their densely packed polymer networks [92,95]. ZnO-based films exhibit low water absorption, ensuring long-term pathogen inhibition by gradual Zn^2+^ ion release, which helps prevent structural collapse when in contact with high-moisture foods. These films have been shown to effectively inhibit *S. enterica* and *S. aureus*, while maintaining food safety with minimal Zn migration [92]. In addition, AgNPs reinforce polymer matrices, forming cross-links that enhance film cohesion, increasing tensile strength and UV-blocking efficiency while exhibiting potent antibacterial activity [96]. The controlled release of Ag⁺ ions ensures long-term stability without excessive leaching, which could weaken the film. Mesoporous NP systems, such as Ag/TiO_2_ composites, regulate ion diffusion, maintaining reinforcement over prolonged exposure [127]. These mechanisms stabilize the film, particularly in high-humidity conditions, where direct food contact can compromise barrier performance. Additionally, AgNP-coated films help retain moisture in fresh produce, preventing dehydration and texture loss [128]. In addition to their structural benefits, Carbon Quantum Dots (CQDs) and Metal–Organic Framework (MOF) contribute intelligent packaging functionalities, such as fluorescence-based spoilage detection and moisture-sensitive response mechanisms. Ezati and Rhim [97] found that CQD-infused pectin films provided UV-blocking and antioxidant activity, while their photoluminescent properties enabled UV-to-blue light conversion, useful for prevention of high-fat foods deterioration. Similarly, MOF-incorporated films, such as MIL-100(Fe)-anthocyanin composites, function as pH-sensitive freshness indicators, detecting ammonia release from meat spoilage, providing a visual indication of food degradation [93].

Their antimicrobial action stems from multiple mechanisms: they release ions that disrupt bacterial membranes, generate reactive oxygen species (ROS) that damage cellular components, and physically interact with microbial cells. They also inhibit biofilm formation and disrupt existing biofilms [129]. Metal nanoparticles exhibit antioxidant effects through several key mechanisms [130,131]: they can donate electrons and transfer hydrogen atoms to neutralize free radicals, effectively stabilizing these reactive species. Additionally, they chelate metal ions like iron and copper, preventing them from catalyzing free radical production. Some nanoparticles also act as catalysts, decomposing harmful reactive oxygen species (ROS) such as hydrogen peroxide. The phenomenon of surface plasmon resonance allows certain nanoparticles to generate localized electric fields that further neutralize free radicals. Moreover, some metal nanoparticles mimic natural antioxidant enzymes, enhancing the breakdown of ROS. They can also directly scavenge various types of ROS and modulate cellular antioxidant systems by inducing the expression of antioxidant enzymes. However, addressing the potential migration of nanoparticles from the packaging into the food and complying with relevant food safety regulations, which may vary by region, are significant drawbacks to consider.

The FDA provides “Guidance for Industry”, listing GRAS substances, but does not guarantee safety at the nanoscale, requiring case-by-case evaluations. Antimicrobial food contact materials must comply with Section 409 of the Federal Food, Drug, and Cosmetic Act and register with the Environmental Protection Agency (EPA) under the Federal Insecticide, Fungicide, and Rodenticide Act (FIFRA). Since 2017, nanomaterials must also meet TSCA reporting requirements. Certain metal oxides, including SnO_2_, CuO, Al_2_O_3_, TiO_2_, and ZnO, are exempt from strict food additive regulations under Threshold of Regulation exemptions for specific uses [132]. Two types of migration limits are established for plastic food contact materials: the overall migration limit (OML) and the specific migration limit (SML). The OML is set at 60 mg of migrating substances per kg of food or food simulants, covering all potential migrants from packaging materials. The SML applies to individual authorized substances and is determined based on their toxicological assessment, considering the acceptable daily intake (ADI) or tolerable daily intake (TDI) established by the European Food Safety Authority (EFSA). The migration limits are calculated under the assumption that a 60 kg person consumes 1 kg of packaged food daily over their lifetime, ensuring safety in accordance with European Union regulations [133].

#### 3.2.4. Organic Acids

The efficacy of organic acids as active compounds stems from their ability to inhibit microbial growth through multiple mechanisms. A significant advantage of organic acids is their regulatory status. Many organic acids are classified as generally recognized as safe (GRAS) by food safety authorities and are approved for use in food applications under European legislation [134]. This regulatory acceptance streamlines their incorporation into food packaging materials, reducing barriers to implementation and accelerating the development of new antimicrobial packaging solutions. The natural origin of organic acids aligns perfectly with the growing consumer demand for clean-label products and sustainable packaging solutions. As common intermediates in living organisms, these acids are inherently biodegradable and environmentally friendly [135]. This characteristic makes them highly compatible with bio-based packaging materials like pectin films, supporting the trend toward more sustainable food packaging options.

The mechanisms of organic acids in active films include antimicrobial activity, where organic acids lower the pH of the food surface, inhibiting the growth of microorganisms and penetrating microbial cell membranes to disrupt cellular functions [116]. pH regulation occurs through the gradual release of organic acids from the film, maintaining a low pH environment that is unfavorable for spoilage and pathogenic microorganisms. Controlled release is facilitated by the pectin film matrix, allowing for the gradual release of organic acids into the food or package headspace over time. Additionally, some organic acids like citric acid exhibit antioxidant properties that help prevent oxidation of food components. All these mechanisms contribute to food preservation by inhibiting microbial growth on the surface, maintaining overall food quality by preventing microbial spoilage and oxidation, and providing a natural alternative to synthetic preservatives. For instance, pectin-based biofilms have been shown to extend the shelf life of various foods, such as seafood (salmon fillet), meat, and fruits (passion fruits), by decreasing oxygen absorption and delaying texture and color changes and the incorporation of organic acids into these films could potentially enhance these preservative effects through additional antimicrobial action [99,100,101].

Examples of organic acids used in pectin-based food packaging include gallic acid, tannic acid, and cinnamic acid, which act through several mechanisms to preserve food quality [99,100,101]. Gallic acid has been observed to enhance polymer cohesion through hydrogen bonding and reinforcing film structure, while its plasticizing effect improves flexibility. Liu et al. [99] reported that gallic acid incorporation increased TS from 6.00 MPa to 15.97–20.06 MPa and EAB from 3.49% to 6.37–7.29%, significantly improving film mechanical properties. The high surface energy of gallic acid–loaded nanoparticles further strengthens interfacial adhesion, ensuring uniform dispersion without voids or cracks, which enhances film ductility and moisture resistance when in direct contact with high-humidity foods like salmon fillets. The incorporation of cinnamic acid into pectin-based films slightly improved mechanical properties, increasing TS to 0.124 ± 0.005 Pa and EAB to 13.882 ± 0.084%, enhancing flexibility for high-moisture applications like beef packaging [100]. More importantly, cinnamic acid–containing films effectively maintained high antimicrobial concentrations by gradually migrating from the packaging material to the product’s surface. This controlled release exhibited strong antimicrobial activity against Gram-positive (*S. aureus*, *MRSA*, *B. subtilis*) and Gram-negative (*Y. enterocolitica*, *P. aeruginosa*, *P. mirabilis*, *S. typhimurium*, *E. coli*, *A. anitratus*) bacteria, extending the shelf life of beef by delaying microbial spoilage [100]. In addition, the incorporation of tannic acid into pectin-based films significantly improves mechanical strength by forming hydrogen bonds and covalent interactions with pectin molecules, creating a denser and more rigid polymer network. Compared to pure pectin films (TS: 12.103 MPa, EAB: 41.59%), the addition of tannic acid increased tensile strength by 40.14%, while further Fe^3+^ incorporation increased it by 42.85% due to the formation of a metal–phenol network (“egg carton” structure), enhancing film durability and structural integrity [101,136]. This improved mechanical strength correlates with the film’s barrier performance, reducing water vapor permeability (WVP) and enhancing its protective effect when applied to perishable foods. When used as a coating for passion fruit, TA-based films significantly reduce moisture loss and delay senescence by limiting water evaporation and modifying anaerobic respiration. Passion fruit treated with PE/TA/Fe^3+^ composite coatings exhibited lower weight loss (~5.98%) over 7 days compared to untreated fruit (~12.17%), effectively reducing wrinkling and spoilage [101].

However, using organic acids in pectin-based active packaging films presents several considerations and challenges, including addressing the potential pH sensitivity of some food products and complying with relevant food safety regulations. For instance, gallic acid is a potent antioxidant but may cause cytotoxic and genotoxic effects at high concentrations. Excessive intake can induce oxidative stress, DNA damage, and apoptosis in normal cells, raising safety concerns [137]. Meanwhile, tannic acid is an FDA-approved food additive recognized for its antioxidant and antimicrobial properties [138]. In India, the daily intake of tannins ranges from 1500–2500 mg, primarily from spices, while in the USA, it averages around 1 g per day. Consumption within 1.5–2.5 g is considered safe, but excessive intake may inhibit iron absorption. Studies indicate that tannins are safe at permissible levels, such as up to 15,000 mg/kg feed for adult ruminants, 10,000 mg/kg for rabbits and laying hens, 1500 mg/kg for pigs, and 1000 mg/kg for chickens, with no reported adverse effects on infants, children, adults, or pregnant women [139]. Cinnamic acid has been evaluated for genotoxicity, reproductive toxicity, respiratory effects, phototoxicity, and environmental safety, with no significant concerns identified. It is not genotoxic, phototoxic, or photoallergenic, and presents no risk for skin sensitization under current usage levels. The NOAEL for repeated dose toxicity is 7.5 mg/kg/day, and for developmental toxicity, it is 50 mg/kg/day. Environmental assessments confirm cinnamic acid is not persistent, bioaccumulative, or toxic (PBT), with risk quotients (PEC/PNEC) below 1, indicating minimal ecological impact [140].

### 3.3. Smart Film Packaging

Color compounds from natural or synthetic sources serve as innovative smart color indicators in packaging films, especially when integrated into pectin-based systems. They offer visual cues about food conditions through color changes triggered by factors such as pH shifts, temperature fluctuations, or specific metabolites from food spoilage. These compounds operate through several mechanisms: firstly, pH sensitivity causes many dyes to change color in response to pH variations common in spoilage [11]; secondly, temperature sensitivity prompts certain compounds to alter their molecular structure with temperature changes [141]; and thirdly, these dyes react with specific spoilage by-products when food spoils, generating metabolites like amines and ammonia [142] from food such as meat, chicken, and seafood, or organic acids from fruits [19]. This interaction occurs through acid-base reactions, altering the dye molecules’ protonation state, inducing oxidation-reduction reactions, and changing its electronic structure in general [143]. These processes collectively result in observable color changes that signal the degree of spoilage in packaged food. Lastly, oxidation sensitivity leads some dyes to change color in response to oxygen exposure, indicating package integrity issues or food exposure to oxygen. These mechanisms enable films to signal freshness visually, warn of spoilage before consumption, indicate temperature misuse for sensitive products, and highlight compromised package integrity.

**Figure 3:**
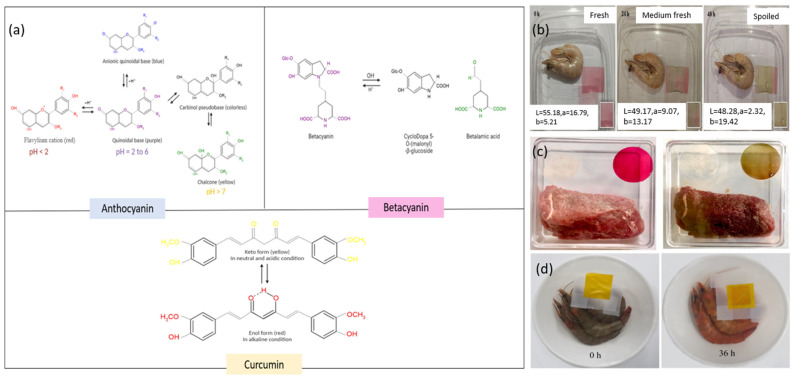
(**a**) pH-dependent mechanism of color change; (**b**) changes in shrimp freshness during storage using intelligent halochromic films (adapted from Tavassoli et al., reprinted with permission from Ref. [144]. Copyright 2023 Elsevier); (**c**) apparent color change of WMP/BTE2 films before and after storage of packaged chilled beef at 4 °C for 8 days (adapted from Guo, Ge et al., reprinted with permission from Ref. [145]. Copyright 2021 Elsevier) (**d**) apparent color change of pectin/curcumin/SNP^3^ film before and after storage of packaged shrimp at 25 °C for 36 h (adapted from Ezati and Rhim, reprinted with permission from Ref. [11]. Copyright 2020 Elsevier).

Natural colorants derived from plant extracts, such as anthocyanins, betacyanins, and curcumin, play a crucial role as smart color indicators in film packaging, providing visual cues about food freshness, pH variations, and exposure to environmental conditions. Recent studies on pectin-based film packaging incorporated with various compounds for smart packaging applications are summarized in Table 4. In addition, the pH-dependent mechanism of color change is illustrated as in Figure 3.

**Table 4:** Pectin-based smart films with natural colorants, color change mechanisms, and food application benefits.

Color Compound Indicator	Pectin Source	Outcome	Application	Reference
Pistachio peel anthocyanins	Commercial pectin	Film color changes: Cherry/pink at pH 1–6 to yellow/brown at pH 9–12	Shrimp	[10]
Purple cabbage anthocyanin	Commercial pectin	Film color changes: Purple at pH 3–6, violet at pH 7 to green at pH 8–11	Chicken breast meat	[93]
Black rice anthocyanin	Commercial pectin	Film color changes: Magenta at pH 1–4, blue at pH 5–12 to chartreuse at pH 13	Meat	[146]
Red cabbage anthocyanin	LM citrus	Film color changes: Red at pH 2, purplish red at pH 3–8, blue at pH 9–11 to greenish yellow at pH 12	Shrimp	[147]
Sumac extract anthocyanin	HM pectin	Anthocyanin color changes: Red at pH 2–4, dark green at pH 8, olive color at pH 9, goldenrod color at pH 10 and 11, to dark goldenrod color at pH 12 Film color changes: Red at pH 7.1 to green-yellow pH 8.06 after 24 h	Shrimp	[144]
Butterfly pea (*Clitoria ternatea*) anthocyanin	HM pectin	Film color changes: Purple at pH 5.8–6.2, blue at pH 6.3–6.6 and green at pH > 6.7	Meat	[148]
Dark red kidney bean (*Phaseolus vulgaris*) anthocyanin	Citron peel pectin	Anthocyanin color changes: Dark purple colour at pH 2–5, light purple at pH 6–7 to yellow-brown pH 8–12 Film color changes: Pink at Day 1, light brown at Day 4 to dark brown at Day 8.	Meat	[149]
Blue honeysuckle berry anthocyanin	Citrus pectin	Film color changes: Purple-dark blue-blue-green-dark green (from pH 6.4 to 8.4) within 24 h	Shrimp	[150]
Pitaya peel betacyanin	Pitaya peel pectin	Betacyanin color changes: Light pink color at pH 2, Red at pH 3–7, light red at pH 8, reddish brown at pH 9, brown at pH 10, yellow at pH 11–12. Film color changes: Light red at Day 0, dark red at Day 4 to brown at Day 8	Shrimp	[151]
Beetroot betacyanin	Watermelon peel pectin	Film color changes: Dark red at pH 3–7, purple at pH 8, yellow pH 9–10. As application in beef packaging, the film color changed from yellow to brown within 8 days of observation.	Beef	[145]
Curcumin	Apple pectin	Yellow at pH 3–8, light red at pH 9–10 to dark red at pH 11–12.	Shrimp	[152]
Curcumin	Citrus pectin	Curcumin color changes: Yellow at pH 4, yellow-orange at pH 5–7, red to dark red at pH 9–12. Film color changes: As application in shrimp packaging, the film color changed from yellow to orange after 36 h of observation.	Shrimp	[11]
Schiff base derived from phenylalanine and syringaldehyde	LM pectin	Film color changes: Orange to brown after 8 days of observation	Fresh-cut mango	[19]

#### 3.3.1. Anthocyanins Compounds

Anthocyanins, a class of flavonoid pigments widely found in the plant kingdom, impart red, purple, and blue hues to various fruits, vegetables, and flowers. Common sources include strawberries, blueberries, blackberries, raspberries, grapes, red cabbage, purple carrots, eggplant, black rice, purple corn, and flowers such as roses, petunias, and hibiscus [153]. The color of anthocyanins varies significantly with pH due to changes in their molecular structure [154,155] as visualized in Figure 3. In acidic conditions (pH < 3), anthocyanins predominantly exist as red-colored flavylium cations, their most stable form. Moving into mildly acidic to neutral conditions (pH 4–6), the flavylium cations convert into colorless carbinol pseudobases, resulting in less intense coloration of anthocyanin-rich solutions. At neutral pH (around 7), anthocyanins adopt quinoidal bases, giving rise to a purple or blue coloration. In alkaline environments (pH > 8), anthocyanins transform further into chalcone structures, which appear yellow [156,157]. This reversible pH-dependent color change allows anthocyanins to serve as dynamic indicators of environmental pH changes.

The stability of pH-sensitive films incorporating anthocyanins is influenced by temperature, time, and light exposure [158]. Higher temperatures and prolonged storage accelerate color degradation, leading to fading, yellowing, or reduced intensity. Studies have shown that at low temperatures (4 °C), pH-sensitive films exhibit gradual color transitions over several days, while at room temperature (25 °C), spoilage-related changes occur much faster, making freshness detection more immediate but also reducing film stability [147,159]. Non-acylated anthocyanins are particularly prone to oxidation, thermal degradation, and structural breakdown, whereas acylated anthocyanins offer greater resistance to heat, pH changes, and enzymatic activity due to steric hindrance [160,161]. Additionally, diglucosides are more stable than monoglucosides, which degrade into phenolic acids and aldehydes over time [162]. Light and oxygen exposure further accelerate oxidative degradation, diminishing the film’s effectiveness in freshness monitoring. To enhance stability, co-pigmentation, encapsulation, and protective polymer matrices can help mitigate environmental effects, ensuring prolonged color integrity and improved reliability in smart packaging applications.

The practical application of anthocyanin-based films in meat and seafood packaging further highlights their pH-dependent behavior. In meat products, the spoilage process is primarily driven by microbial growth, which produces volatile basic nitrogen compounds such as ammonia, trimethylamine, and dimethylamine. These compounds increase the pH of the meat environment, triggering a color change in anthocyanin-based films from red (in acidic conditions) to purple or blue (in alkaline conditions). For instance, studies by [93,146] developed smart packaging films using anthocyanins extracted from purple cabbage and black rice, incorporated into a pectin film matrix. When applied to meat samples, the films changed color from red/purple to blue as the meat spoiled, providing a clear visual indicator of freshness. The color change correlated well with increases in total volatile basic nitrogen (TVB-N) content and microbial growth, demonstrating the film’s effectiveness as a spoilage indicator.

Similarly, anthocyanin-based films have shown significant promise in detecting freshness changes in seafood applications. Seafood products, especially fish and shrimp, are highly susceptible to rapid quality deterioration due to enzymatic and microbial activities. As seafood spoils, it produces volatile amines that increase the pH of the surrounding environment. Studies have demonstrated the effectiveness of pH-sensitive films using anthocyanins extracted from various sources, incorporated into a pectin film matrix, and applied to shrimp [10,144,147]. These films exhibited a gradual color change from red to green/brown as the shrimp spoiled. The color changes correlated well with TVB-N increases, pH, and microbial counts, providing a reliable indicator of shrimp freshness.

#### 3.3.2. Betacyanin Compounds

Betacyanins, which are red-violet pigments from the betalain family found in plants like beets, pitaya, amaranth, prickly pear cactus, Swiss chard, and red quinoa, exhibit an intense red-violet color in their natural state within plant tissues, with betanin from red beets being the most prevalent example of these pigments [163]. The schematic diagram of betacyanin color degradation is visualized in Figure 3. These compounds maintain their red-violet hue across a broad pH range, approximately pH 3–7, which makes them stable under mildly acidic to neutral conditions, enhancing their suitability as natural food colorants. However, when exposed to alkaline conditions (pH > 7), betacyanins degrade, causing a shift in color to brownish-yellow or brown due to the breakdown into yellow betalamic acid and cyclo-DOPA derivatives [164]. Unlike anthocyanins, betacyanins maintain their red-violet color over a broader pH range (approximately 3–7), making them particularly suitable for applications where stability in mildly acidic to neutral conditions is desired. The stability of betacyanins in this pH range is advantageous for packaging fresh food products, as many foods naturally fall within this pH spectrum. For instance, fresh fish typically has a pH between 6.0 and 6.5, allowing a betacyanin-based indicator to maintain its initial red-violet color when the product is fresh.

As seafood begins to spoil, it undergoes various biochemical changes that produce volatile basic nitrogen compounds, primarily trimethylamine (TMA), dimethylamine (DMA), and ammonia. The production of these alkaline compounds causes a gradual increase in pH, which can trigger a color change in betacyanin-based indicators. A study by Jiang et al. [151] developed an intelligent packaging film incorporating betacyanins extracted from red pitaya (dragon fruit) peel. When applied to fresh shrimp samples, the film maintained its original red-violet color during the initial storage period. As the shrimp began to spoil and produce volatile amines, the film gradually changed color to yellow, providing a clear visual indicator of freshness loss. The color change correlated well with increases in TVB-N content and microbial growth, demonstrating the film’s effectiveness as a real-time freshness indicator. The application of betacyanin-based smart packaging extends beyond seafood. For example, [145] developed a betacyanin-incorporated film to monitor the freshness of chilled beef. The film exhibited color changes corresponding to different stages of spoilage, transitioning from pink to brown as the chilled beef deteriorated from fresh to spoiled.

Other than that, betacyanin stability is highly influenced by temperature and exposure time, with higher temperatures accelerating degradation and color loss. Studies show that high temperatures inhibit betacyanin synthesis while low temperatures slow degradation [165]. Thermal processing promotes isomerization, decarboxylation, and cleavage of betanin, leading to a progressive loss of red coloration and the formation of light brown and yellow pigments due to the production of neobetanin and betalamic acid [166]. Extended exposure time also influences degradation, as prolonged heating results in cumulative structural breakdown. Additionally, light exposure further accelerates betacyanin degradation, particularly under UV and visible light, which excites electrons within the pigment structure, making them more prone to breakdown [167]. In contrast, storage in darkness helps maintain pigment integrity, as evidenced by studies where betacyanin-containing films exhibited minimal color change in dark conditions but showed significant fading under continuous light exposure [168,169]. Hence, due to betacyanins degrading faster under light than in dark storage, this has emphasized the need for protective measures such as co-pigmentation, encapsulation, and light-blocking packaging materials to prolong stability.

#### 3.3.3. Curcumin Compounds

Curcumin, a bright yellow compound in the curcuminoid class, is primarily sourced from turmeric (*Curcuma longa*), which contains 2–5% curcumin in its rhizomes. Other Curcuma species like *Curcuma zedoaria* and *Curcuma aromatica* also contain curcumin, albeit in lesser amounts. Within the ginger family (*Zingiberaceae*), some related plants may have trace quantities of curcumin or related compounds [170,171]. The characteristic yellow color of curcumin arises from its molecular structure and interaction with light, featuring two aromatic ring systems linked by a seven-carbon chain with conjugated double bonds that absorb light in the blue-violet region (around 410–430 nm) and reflect yellow light, giving it a bright yellow appearance [172]. The color of curcumin is pH-dependent: in acidic and neutral conditions (pH ≤ 7), curcumin remains yellow, while in alkaline conditions (pH > 7), it shifts to a reddish-brown hue due to deprotonation of its phenolic groups altering its light absorption properties [12]. Curcumin can exist in different tautomeric forms, primarily favoring the enol form in solution, contributing to its stability and color characteristics. The degradation color of curcumin in acidic and alkaline conditions is visualized as in Figure 3.

Curcumin-based smart films are also highly sensitive to storage time and temperature, with degradation primarily occurring through oxidation, hydrolysis, and photodegradation [173,174]. The primary degradation pathways involve oxidative cleavage of the conjugated system, leading to the formation of feruloylmethane, vanillin, and ferulic acid, resulting in loss of yellow coloration and diminished pH sensitivity [175]. Prolonged storage also contributes to light-induced degradation, where UV and visible light excite electrons in curcumin’s chromophore, accelerating photochemical breakdown into non-colored degradation products [176,177].

In the context of smart packaging, curcumin’s pH sensitivity makes it a valuable indicator for food freshness. As foods spoil and become more alkaline, curcumin-based indicators can shift from yellow to reddish-brown, signaling quality issues in packaged foods. Studies on curcumin-incorporated pectin films have shown their effectiveness in monitoring shrimp spoilage [11,152]. Shrimp spoilage involves the breakdown of proteins by enzymes and bacteria, producing volatile basic nitrogen (TVB-N) compounds such as ammonia and trimethylamine (TMA). These compounds increase the shrimp’s pH from slightly acidic (fresh) to alkaline (spoiled). Curcumin-incorporated pectin films initially appear yellow, indicating fresh shrimp’s slightly acidic to neutral pH. As spoilage progresses and pH rises, the curcumin changes color from yellow to orange to reddish-brown, providing a visual indicator of spoilage.

## 4. Conclusions and Future Trends in Pectin-Based Film Packaging

In summary, pectin-based smart and active packaging materials represent a promising approach for enhancing food quality, safety, and shelf-life while reducing waste in the food industry. By incorporating natural pigments and additives such as antimicrobials and antioxidants, these materials can effectively monitor and maintain food freshness through visible color changes in response to environmental factors like pH, gas levels, or temperature shifts. However, developing pectin films with natural extracts poses challenges, including volatility, sensory impact, regulatory compliance, and changes in film properties like transparency and mechanical strength. Addressing issues such as nanoparticle migration, stability of color compounds, and adherence to food safety regulations is crucial for advancing these technologies. Anthocyanin-, betacyanin-, and curcumin-based films are promising but require solutions to ensure stable color retention under varying storage conditions and cost-effective production methods. Strategies like microencapsulation, nanomaterial integration, and digital tracking via QR codes or barcodes are being explored to improve packaging indicators’ sensitivity, specificity, and consumer usability. By overcoming these challenges and continuing to innovate, pectin-based packaging can enable real-time monitoring of food freshness without package opening, offering insights into product history and environmental exposure factors. This ongoing research aims to enhance further the functionality, safety, and sustainability of biodegradable packaging solutions to benefit consumers and the environment.

## Figures and Tables

**Table 3:** Pectin-based active films with incorporation of active compounds, mechanisms, and food application benefits.

Active Compound		Pectin Source	Outcome	Reference
Natural extract	Mulberry leaf extract	Commercial pectin	- Improved mechanical, barrier, antioxidant, and antimicrobial properties. - Increase the shelf life of capsicum up to 12 days.	[84]
	Neem leaf extract	Lemon peel pectin	- Improved the physico-chemical and antimicrobial properties. - Delayed signs of spoilage and increased shelf life of apricot.	[85]
	Thinned young apple polyphenols	Citrus pectin	- Increased thermal stability and mechanical properties. - Good inhibitory effect against *E. coli*, *S. aureus*, and *L. monocytogenes*.	[49]
	Chañar and green tea extracts	Citrus pectin	- Improved mechanical and antioxidant activity properties. - The shelf life of a fatty food package was extended up to 77 days.	[86]
	Green propolis extract	Citrus pectin	- Improved UV-light barrier and antioxidant properties.	[87]
Essential oil	Thyme and oregano essential oil	Commercial pectin	- Increased total phenolic effect and antioxidant activity. - Effective inhibition against all tested bacteria (*E. coli*, *P. aeruginosa*, *S. aureus*).	[88]
	Clove essential oil	Citrus pectin	- Showed inhibition against *S. aureus*, *E. coli*, and *L. monocytogenes* bacteria.	[5]
	*Thymus capitatus* and *Cinnamomum verum* essential oils	Commercial pectin	- Decreased moisture content. - Increased TS and EAB. - High antioxidant and antibacterial activity.	[6]
	*Citrus reticulata* L. (tangerine) essential oil	Commercial pectin	- Decreased WVP, water solubility, and transparency. - Increased DPPH and ABTS antioxidant activity.	[89]
	Grapefruit essential oil	Commercial pectin	- Reduced EAB and transparency. - Increased DPPH• and ABTS•+ radical scavenging activities.	[90]
	Summer savory essential oil	LM pectin	- Inhibited the growth of psychotropic bacteria. - Significantly reduced pH level, TVB-N, and TBA. measurements of chicken fillets during storage period.	[91]
Metal nanoparticles	Silver, zinc, and copper nanoparticles	Commercial pectin	- Antibacterial activity against *E. coli* and *S. aureus.* - Strong antioxidant in ABTS and DPPH.	[4]
	Zinc oxide nanoparticle	Commercial pectin	- Reduced total population of microbes as wrapping in hard cheese sample.	[92]
	MIL-100 (Fe),	Commercial pectin	- Improved tensile strength, thermal stability, barrier performance, and absorption capacity.	[93]
	Pt, Pd, Au, and Ag nanoparticles	Citrus amidated pectin	- Exhibit electrocatalytic properties toward several electrocatalytic reactions.	[94]
	Zinc oxide nanoparticles	Citrus pectin	- Enhanced mechanical and flexibility. - Good antimicrobial activity against pathogens such as *E. coli*, *P. aeruginosa*, *S. enterica Typhimurium*, *L. monocytogenes*, *S. aureus*, and *C. albicans*.	[95]
	Silver nanoparticles	Citrus peel pectin	- Thermal stability, mechanical strength, and water vapor barrier properties of the pectin films increased. - Strong antibacterial activity against food-borne pathogenic bacteria, *E. coli* and *L. monocytogenes*.	[96]
	Carbon quantum dots	Citrus pectin	- Showed potent antioxidant and antimicrobial activities. - Exhibited a light conversion property for UV-blocking.	[97]
	Zinc oxide nanoparticle	Citrus pectin	- Good UV-blocking. - Excellent antibacterial effects against both *E. coli* and *S. Aureus*.	[98]
Organic acids	Gallic acid	Commercial pectin	- Good light barrier and mechanical properties. - Improved antibacterial activity against two common biogenic amine-producing bacteria (*Morganella morganii* and *E. coli*). - Delayed salmon fillets’ biogenic amine generation and extended shelf life for another 3 days.	[99]
	Cinnamic acid	Citrus peel pectin	- Showed significant inhibitory activity on all test foodborne bacteria. - Led to 84.09% bacterial load reduction in beef and improved the colour quality of beef during storage.	[100]
	Tannic acid	Commercial pectin	- Improve the UV barrier properties and antioxidant properties of films. - Reduced weight loss rate and wrinkle index of the passion fruit within 7 days of storage.	[101]
